# Renal Denervation Suppresses Atrial Fibrillation in a Model of Renal Impairment

**DOI:** 10.1371/journal.pone.0124123

**Published:** 2015-04-17

**Authors:** Zhuo Liang, Xiang-min Shi, Li-feng Liu, Xin-pei Chen, Zhao-liang Shan, Kun Lin, Jian Li, Fu-kun Chen, Yan-guang Li, Hong-yang Guo, Yu-tang Wang

**Affiliations:** 1 Department of Cardiology, Chinese PLA General Hospital, Beijing, China; 2 Department of Emergency, Beijing Tsinghua Changgeng Hospital Medical Center, Tsinghua University, Beijing, China; 3 Department of Geriatric Cardiology, Chinese PLA General Hospital, Beijing, China; University Medical Center Utrecht, NETHERLANDS

## Abstract

**Background:**

A close association exists between renal impairment (RI) and atrial fibrillation (AF) occurrence. Increased activity of the sympathetic nervous system (SNS) may contribute to the development of AF associated with RI. Renal denervation (RDN) decreases central sympathetic activity.

**Objective:**

The main objective of the study was to explore the effects of RDN on AF occurrence and its possible mechanisms in beagles with RI.

**Methods:**

Unilateral RI was induced in beagles by embolization of small branches of the renal artery in the right kidney using gelatin sponge granules in Model (n = 6) and RDN group (n = 6). The Sham group (n = 6) underwent the same procedure, except for embolization. Then animals in RDN group underwent radiofrequency ablation of the renal sympathetic nerve. Cardiac electrophysiological parameters, blood pressure, left ventricular end-diastolic pressure, and AF inducibility were investigated. The activity of the SNS, renin-angiotensin-aldosterone system (RAAS), inflammation and atrial interstitial fibrosis were measured.

**Results:**

Embolization of small branches of the renal artery in the right kidney led to ischemic RI. Heart rate, P wave duration and BP were increased by RI, which were prevented or attenuated by RDN. Atrial effective refractory period was shortened and AF inducibility was increased by RI, which were prevented by RDN. Antegrade Wenckebach point was shortened, atrial and ventricular rates during AF were increased by RI, which were attenuated or prevented by RDN. Levels of norepinephrine, renin and aldosterone in plasma, norepinephrine, angiotensin II, aldosterone, interleukin-6 and high sensitivity C-reactive protein in atrial tissue were elevated, and atrial interstitial fibrosis was enhanced by RI, which were attenuated by RDN.

**Conclusions:**

RDN significantly reduced AF inducibility, prevented the atrial electrophysiological changes in a model of RI by combined reduction of sympathetic drive and RAAS activity, and inhibition of inflammation activity and fibrotic pathway in atrial tissue.

## Introduction

Patients with chronic kidney disease (CKD) show a high prevalence of atrial fibrillation (AF) [1,2,3]. Exploring the inherent pathogenic mechanisms responsible for the development of AF among CKD patients and identifying effective therapeutic targets are urgent. CKD is accompanied by ischemic renal impairment (RI) and renal dysfunction. The ischemic RI lead to increased sympathetic activation [4]. Hyper-sympathetic activity is involved in atrial remodeling processes [5,6]. The renin-angiotensin-aldosterone system (RAAS) is also activated by renal ischemic impairment and increased sympathetic activation in CKD [7], and angiotensin II and aldosterone create a substrate for AF [8,9]. Previously, we showed that AF was associated with RI with mild renal insufficiency and increased activity of the sympathetic nervous system (SNS) and RAAS may contribute to the development of AF associated with RI with mild renal insufficiency in animal experiment [10]. However, intervention measures to inhibit the activity of SNS or RAAS need to be further applied. It will be important for elucidating mechanisms and developing new therapeutic strategies for CKD-induced atrial arrhythmogenic remodeling.

We hypothesized that modulation of the SNS might reduce AF susceptibility in CKD. Renal denervation (RDN), which is a new therapeutic approach to treat resistant hypertension through reducing renal norepinephrine spillover, can also reduce susceptibility to AF in animal models of obstructive sleep apnea and heart failure by reduction of sympathetic drive, RAAS activity and atrial fibrosis [11,12,13]. However, the effect of RDN after RI on AF inducibility is unknown. In this study, unilateral diffuse ischemic RI was induced in dogs by transcatheter embolization of small renal artery branches using gelatin sponge granules. By treating dogs with RDN, the role of sympathetic activation for AF vulnerability associated with RI was specifically addressed. Effects of RDN on RAAS activation, atrial inflammation and fibrosis in dogs with RI were also explored.

## Methods

### Ethics statement

This study was carried out in strict accordance with the recommendations in the Guide for the Care and Use of Laboratory Animals of the National Institutes of Health (Publication No. 85–23, revised 1996). The protocol was approved by the Institutional Animal Care and Use Committee of the Chinese PLA General Hospital.

### Experimental animals

The experimental animals included 18 healthy, 5-year-old beagles weighing 10–12 kg. All dogs were anesthetized with intravenous sodium pentobarbital (20 mg/kg) and were intubated using an endotracheal tube and mechanical ventilation. Heart rate and rhythm were monitored by a continuous 3-lead electrocardiogram. A 6F sheath was placed in the right femoral artery. Systolic blood pressure (SBP) and diastolic blood pressure (DBP) were monitored via the sheath using an invasive blood pressure (BP) monitor. A pigtail catheter was introduced into the left ventricle (LV) through the arterial sheath to detect LV end-diastolic pressure (LVEDP).A bolus of heparin (4000 IU) was administrated through the sheath to prevent thromboembolism.

### Experimental design

At baseline, an electrocardiogram, BP and LVEDP were monitored. Electrophysiological examinations were performed. Plasma parameters were measured. Then dogs were divided into three groups. Group 1 (n = 6) served as a control (Sham). In group 2 (n = 6), RI was induced (Model). In group 3 (n = 6), RDN was performed after RI (RDN). After 2 weeks of feeding, the same parameters measured at baseline were measured and renal artery angiography was performed again. Creatinine clearance (CCr) was determined by 30-min endogenous creatinine clearance method [14]. Dogs were then sacrificed humanely by an intravenous overdose of thiopental (2 g). Hearts and right renal arteries were removed for further histology analysis.

### Experimental model for RI

A 5F multifunction catheter was introduced through the arterial sheath and renal artery angiography was performed under fluoroscopy. Following renal artery angiography, RI was induced in dogs by transcatheter embolization of small branches of the right renal artery using gelatin sponge granules (diameter ~50 μm, 30–40mg), whereas the main renal artery or sub-segment renal artery was kept fluent [10]. In the Sham group, normal saline was injected into the renal artery after renal artery angiography as a sham procedure.

### Catheter-based RDN

In the RDN group, a 6F ablation catheter (Biosense Webster, Inc., Diamond Bar, CA, USA) was introduced into the right renal artery via the femoral artery. The tip of the catheter was positioned under direct vision to ensure that it was placed accurately in the renal artery. The renal artery was divided into four areas, from the ostia to the area near the bifurcation based on angiographic imaging. Each discrete radiofrequency ablation (6 watts) lasted up to 80 s, for a total four ablations longitudinally and circumferentially within the four areas of the right renal artery [15]. In the Sham and Model groups, the catheters were introduced into the right renal artery, without ablation being performed.

### Electrophysiological examinations

The right femoral vein was cannulated for catheter insertion. The tip of a multielectrode catheter was placed on the lateral right atrium to record right atrial potentials and to induce rapid atrial pacing. A train of eight basic stimuli (S1, pulse duration 1 ms) at twice the diastolic pacing threshold was followed by an extra stimulus (S2). The atrial effective refractory period (AERP) was defined as the longest S1S2 interval that failed to elicit a propagated atrial response. The AERP was measured at basic pacing cycle lengths of 300 ms and 240 ms, and the S1–S2 intervals were decreased from 200 ms to refractoriness by decrements of 5 ms (LEAD-7000, multi-channel physiology recorder; Sichuan Jinjiang Electronic Science and Technology Co., Ltd, Sichuan, China). The longest cycle length of atrial pacing causing second-degree atrioventricular nodal block (antegrade Wenckebach point) was determined. After the AERP and antegrade Wenckebach point were determined, rapid atrial pacing (60 ms of basic cycle lengths, 10 s in duration, four-fold threshold current) was delivered for 10 times to induce AF (DF-5A, heart stimulator; Suzhou Dongfang Electronic Instruments Plant, Jiangsu, China). AF was defined as irregular atrial rates (cycle length <200 ms; duration >5 s) with irregular atrioventricular conduction. AF inductibility was defined as (the relative ratio of successful induction frequency to total frequency of pacing in each group) ×100%. All AA-and RR-intervals during AF were calculated to determine the mean atrial and ventricular rates during AF.

### Plasma measurements and urinalysis

Blood samples were collected from the femoral vein into tubes containing EDTA, and immediately centrifuged at 2310× g for 10 minutes at 4°C, and then finally stored at -80°C until further assay. Levels of norepinephrine, renin, aldosterone and creatinine in plasma and creatinine in urine were examined by ELISA (Wuhan Beinglay Biotech Co., Ltd., Hubei, China).

### Histologic studies

Renal arteries with their perivascular tissues were removed and immediately dissected and fixed in formaldehyde. A month later, the renal arteries with their perivascular tissues were stained with hematoxylin-eosin (HE) to observe the structure of the renal arteries and renal sympathetic nerves in the three groups. The renal arteries with their perivascular tissues were cut into four parts transversely from the ostia to the area near the bifurcation. Each part was fixed, embedded in paraffin, and sliced into sections (5 μm thickness) of which 10 were taken from each part and processed. All sections were stained with H&E. Sections from each part with the most injured nerves were examined and the total number of nerves and the number of injured nerves were recorded. The distance from the nerves to the renal artery lumen-intima interface was also measured. Left atria were carefully removed. Part of atrial tissue was fixed in 10% phosphate-buffered formalin, and embedded in paraffin. Deparaffined sections (5 μm thickness) were stained with Massontrichrome. Connective tissue was differentiated on the basis of its color (blue) and expressed as a percentage of the reference tissue area using Image-Pro Plus 4.5. In each atrium, 3 images with a magnification of ×400 were analyzed and averaged. Part of atrial tissue was stored at -80°C until further assay. Levels of norepinephrine, angiotensin II, aldosterone, interleukin-6 (IL-6) and high sensitivity C-reactive protein (hs-CRP) in atrial tissue were examined by ELISA (Wuhan Beinglay Biotech Co., Ltd., Hubei, China).

### Statistical analysis

Values are shown as mean ± SD. For comparisons of single repeatedmeasures only, a paired Student t test was used. Differences of the changes in value between two weeks and the baseline among the three groups were subjected to ANOVA followed by the Dunnet test. ANOVA was also used followed by the Dunnet test to compare differences of norepinephrine, angiotensin II, aldosterone, IL-6, hs-CRP and interstitial fibrosis in atrial tissue and CCr among the three groups. The chi-square test was used to compare the AF induction rate. P<0.05 was considered statistically significant.

## Results

### Establishment of the Model of RI and RDN

**Fig 1:**
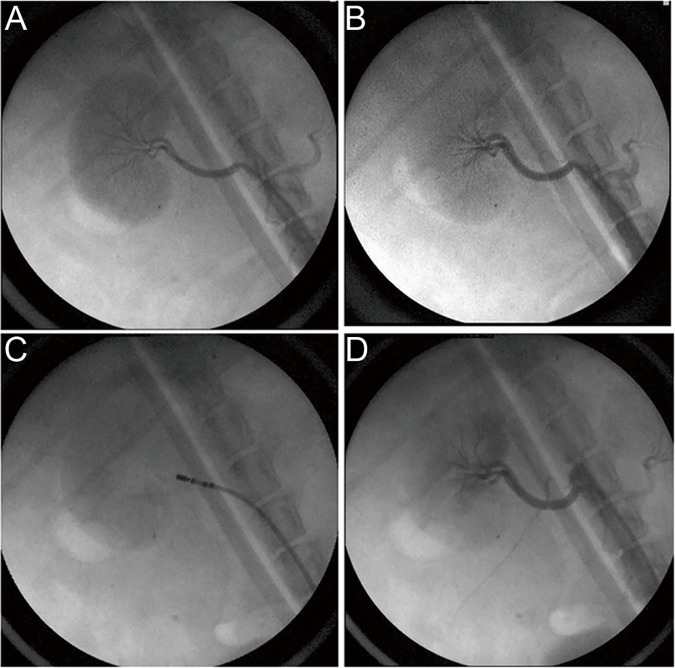
Fluoroscopic images of the right renal artery. Images of the right renal artery angiography before transcatheter embolization (A) and after transcatheter embolization (B) in RDN group. Images of ablation catheter which was placed in the right renal artery after renal impairment for ablation (C). Images of the right renal artery angiography after 2 weeks of ablation (D).

**Fig 2:**
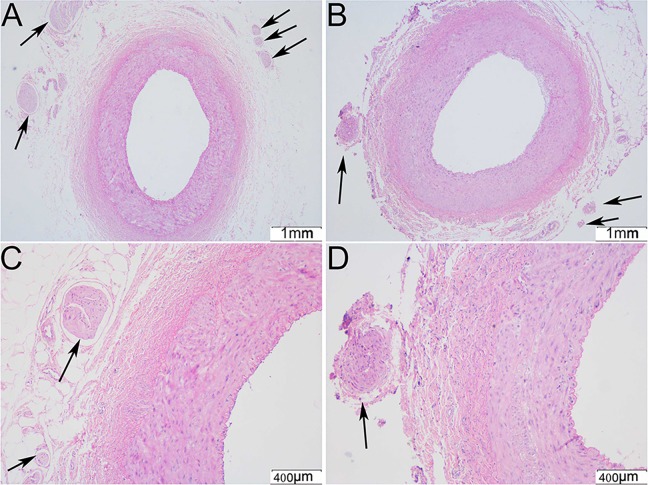
Hematoxylin-eosin (HE) staining images of the right renal artery and renal sympathetic nerves. Images of HE staining of the right renal artery and renal sympathetic nerves (arrow indicated) with a magnification of ×40 in the Model (A) and RDN (B) groups. Images of HE staining of the renal sympathetic nerves (arrow indicated) with a magnification of ×100 in the Model group (C) and RDN groups (D).

Right renal artery angiography was performed before (Fig 1A) and after (Fig 1B) transcatheter embolization in the RDN group. Small renal artery branches were occluded after transcatheter embolization, whereas the main renal artery or sub-segment renal artery remained fluent. Fig 1C shows representative images of ablation using 6F ablation catheter in the right renal artery after RI. The right renal artery had no obvious stenosis after 2 weeks of ablation (Fig 1D). The CCr in the Model and RDN groups were slightly decreased by 22.2% and 26.4% separately compared with the Sham group after 2 weeks of RI (P < 0.05 for each). There was no difference of CCr between the Model and RDN groups. Fig 2 shows representative images of HE staining of the right renal artery and renal sympathetic nerves (arrow indicated) with different magnification in the RDN (B, D) and Model (A, C) groups respectively. Renal sympathetic nerves were intact and surrounded by outer membrane in the Model group. On the contrary, renal sympathetic nerves were damaged in the RDN group, which had contracted morphology and lost surrounding outer membrane. Approximately 49% of renal nerves observed exhibited injury due to the RDN procedure (n = 96 of 196). More than 90% of renal nerves were found between 1.0–3.0mm from the renal artery lumen-intima interface and about 90% of the injured nerves were found in this range. The impedance decreased from 252.5±32.5Ω to 233.8±30.6Ω (P < 0.05, n = 24) after ablation in the RDN group.

### Effects of RDN on ECG, BP and LVEDP

**Fig 3:**
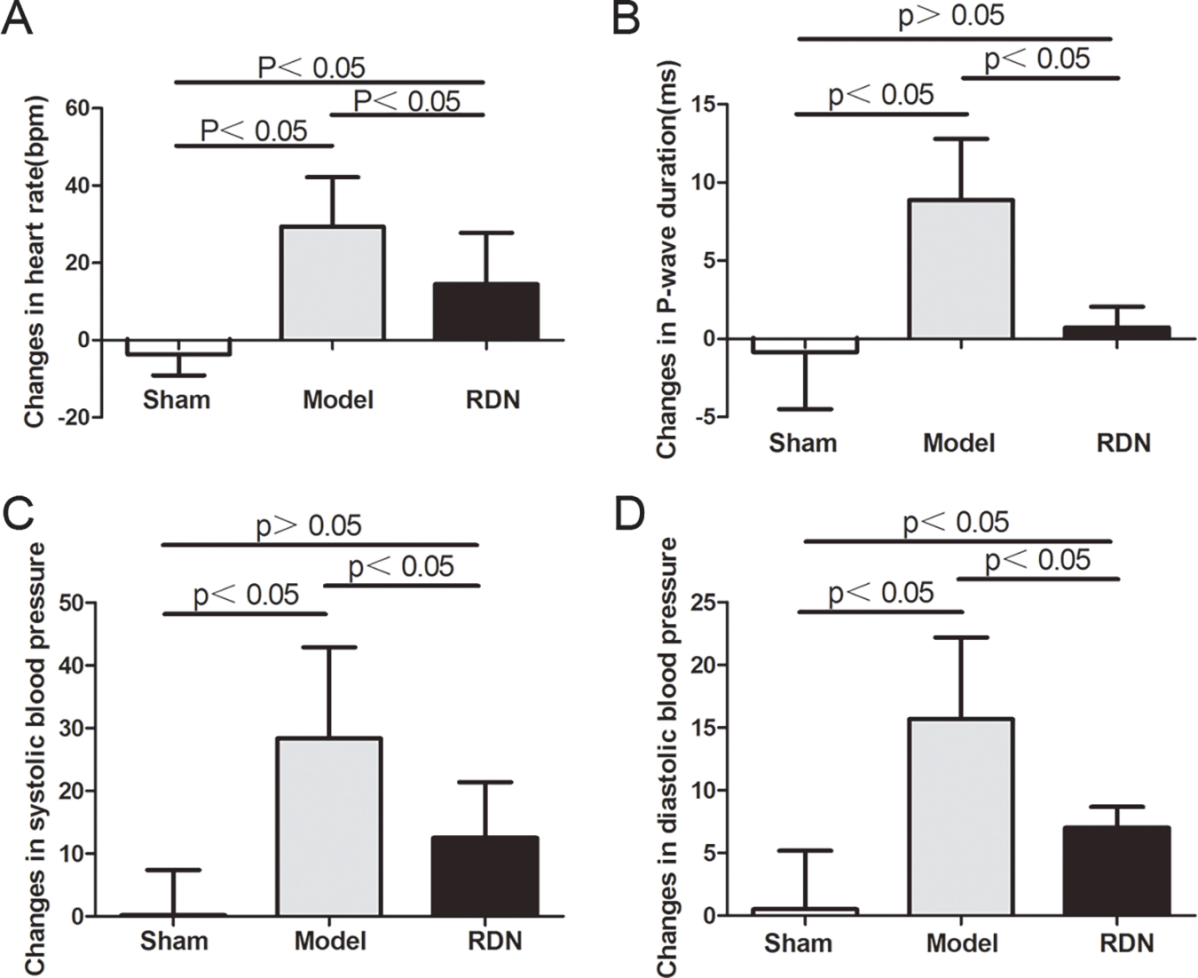
ECG and blood pressure analysis (n = 6). Effects of RDN on the RI-induced heart rate (A), P wave duration (B), systolic blood pressure (C) and diastolic blood pressure (D) changes between values of 2 weeks and baseline.

Sham operation did not change the heart rate, P wave duration, systolic and diastolic blood pressure after 2 weeks, compared with the baseline values in the Sham group. In contrast, 2 weeks of RI induced a pronounced increase in heart rate, P wave duration, systolic blood pressure and diastolic blood pressure by 22.5%, 12.9%, 18.2% and 16.4% respectively, compared with the baseline values in the Model group (P < 0.05 for each). RDN reduced RI-induced heart rate increase, systolic blood pressure increase and diastolic blood pressure increase by 50.5%, 55.8% and 55.4%, respectively (P < 0.05 for each) (Fig 3A, 3C and 3D). RDN completely prevented RI-induced P wave duration increase (P < 0.05) (Fig 3B). LVEDP was unchanged significantly after 2 weeks, compared with the baseline values in the three groups.

### Effects of RDN on AERP and AF Inducibility

**Fig 4:**
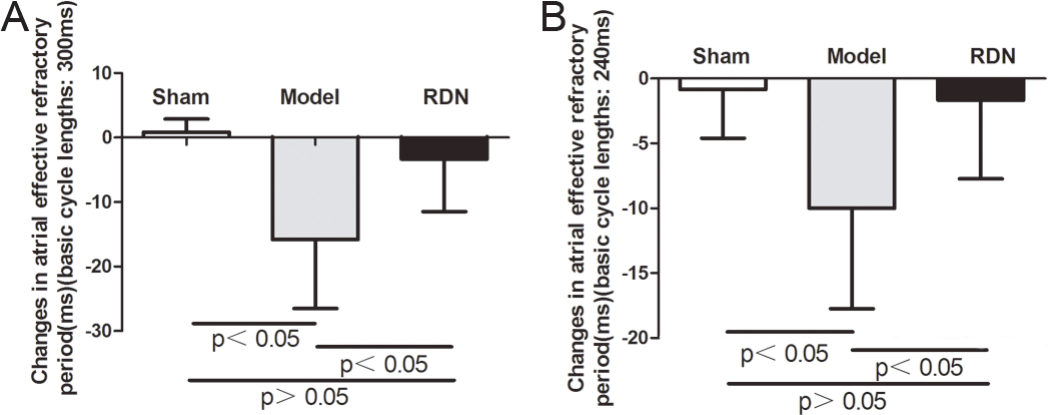
Atrial effective refractory period (AERP) analysis (n = 6). Effects of RDN on RI-induced AERP changes between values of 2 weeks and baseline at 300-ms basic cycle lengths (A) and at 240-ms basic cycle lengths (B).

**Fig 5:**
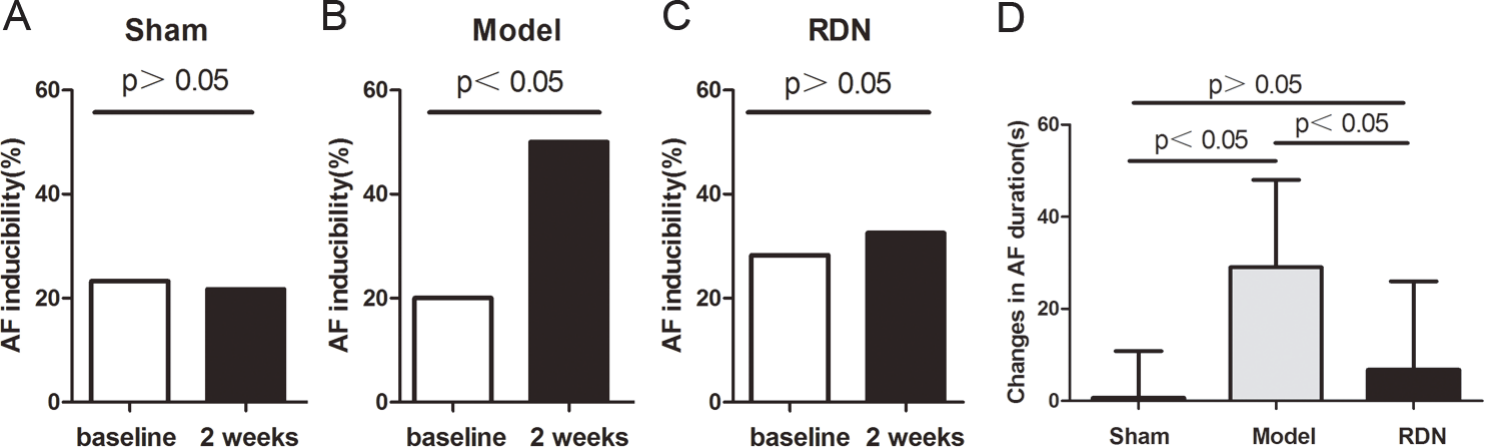
Analysis of the occurrence of AF (n = 6). The inducibility of AF in the Sham (A), Model (B) and RDN (C) groups at baseline and 2 weeks. Effects of RDN on RI-induced changes of AF duration between values of 2 weeks and baseline (D).

Sham operation did not change the AERP at 300-ms or at 240-ms basic cycle lengths after 2 weeks, compared with the baseline values in the Sham group. In contrast, 2 weeks of RI induced a pronounced AERP shortening at 300-ms basic cycle lengths and at 240-ms basic cycle lengths by 10.8% and 7.4% respectively, compared with the baseline values in the Model group (P < 0.05 for each). RDN completely prevented RI-induced AERP shortening at 300-ms basic cycle lengths and at 240-ms basic cycle lengths (P < 0.05 for each) (Fig 4A and 4B). Sham operation did not change the inducibility (Fig 5A) or duration of AF after 2 weeks, compared with the baseline values in the Sham group. In contrast, 2 weeks of RI resulted in a significant increase in AF inducibility by 1.5 fold (P < 0.05) (Fig 5B) and prolonged the duration of AF by 1.86 fold compared with the baseline values in the Model group (P < 0.05). The AF inducibility was unchanged after 2 weeks, compared with the baseline values in the RDN group (P > 0.05) (Fig 5C). RDN completely prevented RI-induced prolongation of AF duration (P < 0.05) (Fig 5D).

### Effects of RDN on Antegrade Wenckbach Point, atrial and ventricular rates during AF

**Fig 6:**
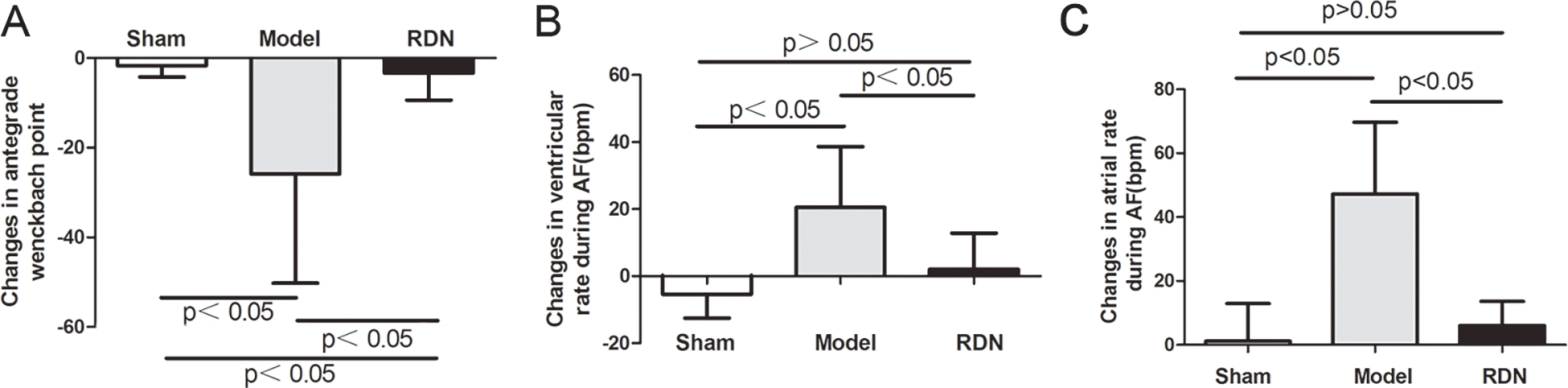
Analysis of the Antegrade Wenckebach point, atrial and ventricular rates during AF (n = 6). Effects of RDN on the changes of Antegrade Wenckbach Point (A), ventricular (B) and atrial (C) rates during AF between values of 2 weeks and baseline.

Sham operation did not change the Antegrade Wenckbach Point, arial or ventricular rates during AF after 2 weeks, compared with the baseline values in the Sham group. In contrast, 2 weeks of RI induced a pronounced shortening of Antegrade Wenckbach Point by 11.2%, a significant increase in atrial and ventricular rates during AF by 11.4% and 9.8% respectively, compared with the baseline values in the Model group (P < 0.05 for each). RDN reduced RI-induced Antegrade Wenckbach Point shortening by 87.2% (Fig 6A), and completely prevented RI-induced increase in ventricular and atrial rates during AF (P < 0.05 for each) (Fig 6B and 6C).

### Effects of RDN on the activity of SNS

**Fig 7:**
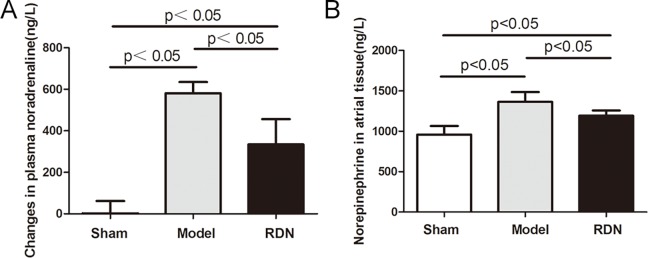
Analysis of the activity of SNS. Effects of RDN on the changes of plasma noradrenaline levels between values of 2 weeks and baseline (A) (n = 6). Left atrial noradrenaline levels in the three groups (B) (n = 6).

Plasma noradrenaline levels were measured to represent systematic activity of SNS. Sham operation did not change the plasma noradrenaline levels after 2 weeks, compared with the baseline values in the Sham group. In contrast, 2 weeks of RI induced a significant increase in plasma noradrenaline levels by 71.6%, compared with the baseline values in the Model group (P < 0.05). RDN reduced RI-induced increase in plasma noradrenaline levels by 42.5% (P < 0.05) (Fig 7A). Levels of noradrenaline in left atrial tissue were measured (Fig 7B). Compared with the Sham group, levels of noradrenaline were increased in the Model group by 42.3% (P < 0.05). In the RDN group, levels of noradrenaline were reduced by 12.6%, compared with the Model group P < 0.05).

### Effects of RDN on the Activity of RAAS

**Fig 8:**
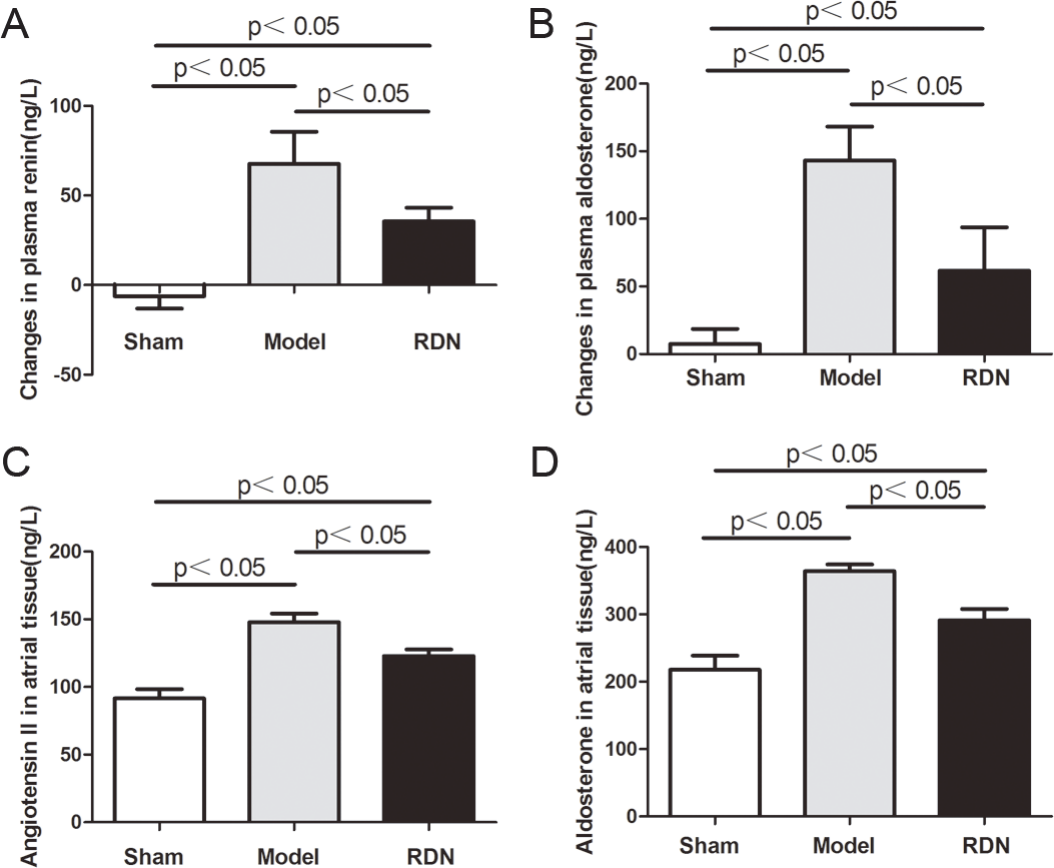
Analysis of the activity of RAAS. Effects of RDN on the changes of plasma levels of renin (A) and aldosterone (B) between values of 2 weeks and baseline (n = 6). Left atrial angiotensin II (C) and aldosterone (D) levels in the three groups (n = 6).

Sham operation did not change the plasma levels of renin and aldosterone after 2 weeks, compared with the baseline values in the Sham group. In contrast, 2 weeks of RI induced a pronounced increase in plasma levels of renin and aldosterone by 56.2% and 59.9% respectively, compared with the baseline values in the Model group (P < 0.05 for each). RDN reduced RI-induced increase in plasma levels of renin and aldosterone by 47.3% and 57.1% respectively (P < 0.05 for each) (Fig 8A and 8B). Levels of angiotensin II and aldosterone in left atrial tissue were shown in Fig 8C and 8D, respectively. Compared with the Sham group, left atrial tissue levels of angiotensin II and aldosterone were increased by 61.3% and 67.1% respectively in the Model group (P < 0.05 for each). In the RDN group, levels of angiotensin II and aldosterone were reduced by 16.9% and 20% respectively, compared with the Model group (P < 0.05 for each).

### Effects of RDN on the inflammation

**Fig 9:**
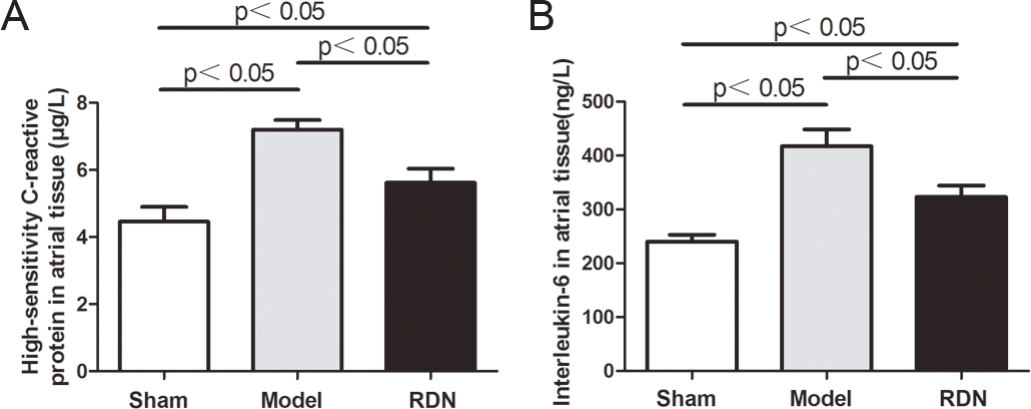
Analysis of the activity of left atrial inflammation (n = 6). Left atrial high sensitivity C-reactive protein (A) and interleukin-6 (B) levels in the three groups.

Left atrial tissue levels of hs-CRP and IL-6 were measured to represent the activity of inflammation (Fig 9A and 9B). Compared with the Sham group, levels of hs-CRP and IL-6 were increased by 61.4% and 73.8% respectively in the Model group (P < 0.05 for each). In the RDN group, levels of hs-CRP and IL-6 were reduced by 21.9% and 22.6% respectively, compared with the Model group (P < 0.05 for each).

### Effects of RDN on the atrial fibrosis

**Fig 10:**
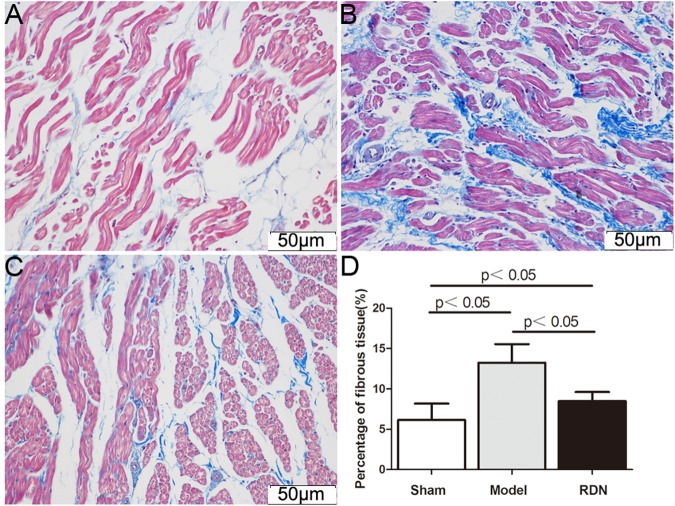
Analysis of atrial fibrosis. Representive images of Masson staining of the left atrial tissue after 2 weeks of interventional operation in the Sham (A), Model (B) and RDN (C) groups. Mean percentage of interstitial fibrosis of the left atrium in the three groups (D) (n = 6).

Fig 10A, 10B and 10C illustrate representive images of Masson staining of the left atrial tissue after 2 weeks of interventional operation in the Sham, Model and RDN groups, respectively. The quantitative ratio of the area of interstitial fibrosis was summarized in Fig 10D. Compared with the Sham group (6.1±2.0%), extensive and heterogeneous interstitial fibrosis was observed in the Model group (13.3±2.3% P < 0.05). The interstitial fibrosis induced by RI was attenuated in the RDN group (8.5±1.1% P < 0.05).

## Discussion

This is the first reported experiment demonstrating that RDN could suppress RI-associated AF occurrence. RDN also attenuated the RI-induced increase in SNS and RAAS activities as well as atrial inflammation and fibrosis, which might contribute to AF occurrence in an RI model. To date, only two animal studies have shown that RI is associated with the development of AF [10,16]. A classic model of CKD was created in rats with 5/6 nephrectomy in which oxidative stress might have been involved in the pathogenesis of interstitial fibrosis and enhanced vulnerability to AF in the left atrium [16]. We also established a large-animal model after 2 weeks of ischemic RI with mild renal insufficiency that was associated with vulnerability to AF [10]. In addition to the mechanism of oxidative stress, inflammation and RAAS and SNS activation are predicted to play important roles in the development of CKD-associated AF [17]. To date, there have not been any published experimental studies to clarify these possible mechanisms because of the lack of appropriate animal models.

Renal denervation is used to reduce renal norepinephrine spillover. It inhibits pronounced shortening of the AERP and reduced susceptibility to AF in animal models of obstructive sleep apnea and heart failure [11,12] by combined reduction of sympathetic drive and RAAS activity [13,15].

In this study, we established an in vivo model of RI that is associated with vulnerability to AF in a large animal (dogs). RDN was applied to demonstrate further that increased sympathetic activation played an important role in RI-induced AF. RDN may be a promising therapeutic strategy for CKD-induced atrial arrhythmogenic remodeling.

Accumulating evidence has shown that in injured kidneys afferent signals to central integrative structures in the brain lead to increased sympathetic activation. Also, the sympathetic nervous system plays an important role in the pathophysiology and progression of CKD [18]. RDN could completely normalize atrial systolic and diastolic pressure, the plasma norepinephrine level, and the heart rate, which had been increased by renal injury in a rat model [19]. RDN reduced the RI-induced increase in the plasma norepinephrine level, heart rate, and blood pressure significantly in our study, which is in accord with the results of a previous study. LVEDP had not changed significantly after 2 weeks compared with baseline values in any group in our present study. Our previous study also showed that hypertension did not affect left atrial pressure, and its effect on vulnerability to AF was negligible in our model [10]. The effect of RDN on hypertension in our model, however, was still important. Although the effect of RDN on resistant hypertension is still debatable [20], RDN may provide a new therapeutic strategy for CKD-induced hypertension because this kind of hypertension is closely related to the hypersympathetic activity induced by RI.

Previous studies have provided evidence that sympathetic activity is involved in the initiation and maintenance of AF [5]. Reducing cardiac sympathetic outflow by cryoablation of the bilateral stellate ganglia and T2–T4 thoracic ganglia can effectively eliminate paroxysmal atrial tachyarrhythmia in dogs with pacing-induced heart failure [21]. In our study, AF inducibility was unchanged after 2 weeks compared with the baseline values in the RDN group, and RDN completely inhibited RI-induced prolongation of AF duration. These results further demonstrated that sympathetic activity plays an important role in AF associated with RI.

Overactivity of the SNS might play an important role in shortening the effective refractory period because adrenergic stimulation alone can decrease the human AERP by approximately 5% [22]. Although a previous study showed that RDN did not significantly influence AERP in normal pigs [23], RDN completely inhibited RI-induced AERP shortening and RI-induced increase in the atrial rate during AF in our study. These data predicted that RDN might influence atrial electrical remodeling only when the autonomic nervous balance is disrupted. AERP shortening induced by sympathetic activity in our RI model may contribute to AF occurrence associated with RI.

It is well known that sympathetic overactivity shortens the antegrade Wenckbach point and accelerates AV conduction, whereas vagal stimulation has the opposite effect [24]. There has been a report of reduced ventricular heart rate in a patient with permanent AF undergoing RDN. Also, RDN has been reported to prolong the antegrade Wenckbach point and provided rate control during AF in pigs [23]. In our study, RDN reduced RI-induced antegrade Wenckbach point shortening and completely inhibited any RI-induced increase in atrial and ventricular rates during AF. Our data indicate that RDN could be promising strategy for rate control in RI-associated AF.

It is also well known that renal sympathetic stimulation induces renin release. The RAAS can be activated by renal ischemic impairment and increased sympathetic activation in CKD [7]. The RAAS is involved in myocardial inflammation and fibrosis and creates a substrate for AF [8,25]. In our study, RDN attenuated RAAS activation induced by RI in both the circulating system and atrial tissue. RDN also attenuated atrial tissue inflammation and fibrosis induced by RI and completely inhibited any increase in RI-induced P-wave duration. These results indicated that the RAAS activated by increased sympathetic activation may also contribute to RI-associated AF occurrence. The above data predicted that the hypersympathetic activity may be a trigger that facilitates initiation of RI-associated AF and may be indirectly involved in the activation of RAAS, atrial inflammation, and fibrosis.

### Study limitations

The pathophysiological process and severity of renal impairment in our model is not completely in accord with the real situation of CKD. Although RDN was applied immediately after RI in our study, there should be a broader time window after RI and before RDN application in future studies. Also, the effect of RDN on AF inducibility, atrial electrophysiological changes, RI-induced hypertension, and renal function in our model needs to be assessed over the long term.

### Conclusions

We established a canine model of RI with mild renal insufficiency. Using this model, we showed that hypersympathetic activity may facilitate the initiation of RI-associated AF because RDN significantly reduces AF inducibility and shortens its duration. We also showed in our RI model that hypersympathetic activity may be involved directly in atrial electrophysiological changes and indirectly in the activation of RAAS, atrial inflammation, and fibrosis. RDN may provide a new therapeutic strategy for CKD-induced atrial arrhythmogenic remodeling and hypertension.
