# Correction: Renal Denervation Suppresses Atrial Fibrillation in a Model of Renal Impairment

**DOI:** 10.1371/journal.pone.0131366

**Published:** 2015-06-18

**Authors:** The PLOS ONE Staff

Author Hong-yang Guo should be listed as the co-corresponding author. Hong-yang Guo may be contacted through email at guohongyang301@126.com.
